# Distinct Endothelial Phenotype Associates with Macrophage-Enriched Microenvironments in Triple-Negative Breast Cancer

**DOI:** 10.21203/rs.3.rs-8595402/v1

**Published:** 2026-01-23

**Authors:** L Becerra-Dominguez, L Yu, CH Rivas, S Aguirre, DG Edwards, I Hu, L Wu, T Lee, X Hao, F Liu, Xiang H.-F. Zhang

**Affiliations:** 1 Lester and Sue Smith Breast Center, Baylor College of Medicine, Houston, TX, USA; 2 Dan L. Duncan Cancer Center, Baylor College of Medicine, Houston, TX, USA; 3 Department of Molecular and Cellular Biology, Baylor College of Medicine, Houston, TX, USA; 4 McNair Medical Institute, Baylor College of Medicine, Houston, TX, USA; 5 Graduate Program in Immunology and Microbiology, Baylor College of Medicine, Houston, TX, USA; 6 Graduate Program in Cancer and Cell Biology, Baylor College of Medicine, Houston, TX, USA; 7 Graduate Program in Quantitative and Computational Biosciences, Baylor College of Medicine, Houston, TX, USA; 8 St. Agnes Academy, Houston, TX, USA

**Keywords:** Triple-Negative Breast Cancer, Macrophages, Vascular Normalization, Endothelial Cells, Tumor Microenvironment

## Abstract

Triple-negative breast cancer (TNBC) is an aggressive malignancy with limited therapeutic options due to the absence of targetable receptors and pronounced intra-tumoral heterogeneity. Among the various contributors to this heterogeneity, the aberrant tumor vasculature plays a critical role by restricting drug delivery and immune cell infiltration, thereby promoting therapeutic resistance. Using previously established murine TNBC models differing in macrophage abundance, we found marked differences in tumor vasculature between these models. Macrophage-enriched tumors exhibited hallmarks of vascular normalization, including increased pericyte coverage and enhanced lectin-based vessel perfusion compared to macrophage-poor tumors. Single-cell RNA sequencing revealed that endothelial cells from macrophage-enriched tumors upregulated inflammatory and macrophage–monocyte interaction genes, including interferon-γ–responsive pathways, and were enriched for venous-like states. In contrast, non-macrophage-enriched tumors displayed arterial-, lymphatic-, and homeostatic endothelial programs. Analysis of human TNBC single-cell datasets confirmed that endothelial composition and activation state vary with macrophage abundance, with macrophage-enriched tumors exhibiting more immunologically active endothelium. Collectively, these results identify conserved associations between macrophage-enriched microenvironments and vascular states, highlighting coordinated immune–vascular dynamics in TNBC tumors.

## Introduction

Breast cancer (BC) remains a leading cause of cancer-related death among women, with an estimated 42,170 deaths projected in the United States in 2025 [1]. Among all BC subtypes, triple-negative breast cancer (TNBC) is the most aggressive, with a five-year survival rate of only 77% [2]. This poor outcome largely reflects the absence of hormone receptors (ER, PR) and HER2 amplification, thereby limiting the applicability of targeted therapies used in other breast cancer subtypes [3].

Beyond its lack of receptors, increasing evidence shows that TNBC encompasses highly heterogeneous intratumoral microenvironmental states, each differing in immune composition, stromal organization, and therapeutic sensitivity [4–9]. A deeper understanding of TNBC heterogeneity, particularly within the tumor microenvironment (TME), is therefore critical and may uncover new mechanisms for treatment that would improve outcomes for TNBC patients.

Prior work from our group identified murine TNBC models that classify into macrophage-enriched (designated as High-MAC in this study) and neutrophil-enriched but non-macrophage-enriched (designated as Low-MAC) immune microenvironments with distinct responses to therapy [10–12]. While these studies revealed the breadth of immune heterogeneity in TNBC, how such immune states relate to other aspect of the TME, such as vasculature, remains poorly understood.

Vascular dysfunction is a hallmark of cancer [13], limiting drug delivery and immune infiltration and efforts to block angiogenesis directly have achieved limited success, shifting attention to vascular normalization as a therapeutic strategy [14–19]. Yet, whether distinct immune microenvironments in TNBC coincide with different vascular states has not been systematically evaluated. Prior studies have shown bidirectional crosstalk between pericytes, endothelial cells, and immune cell, where changes in vascular normalization can influence immune infiltration and vice versa [20].

Here, we propose that TNBC heterogeneity influences not only immune composition but also the vascular compartment. Specifically, we hypothesize that distinct immune microenvironments, particularly those differing in macrophage abundance are associated with unique vascular states, potentially reflecting differences in vascular normalization, and immune–vascular communication.

Using multiple murine models representing High-MAC and Low-MAC states [10–12], we compared vessel normalization and profiled endothelial cell heterogeneity by single-cell RNA sequencing. We then extended these observations to a large human breast cancer single-cell dataset [21] to determine whether the associations identified in mice are conserved in patients. Together, these studies reveal that vascular states in TNBC vary with immune context, highlighting immune–vascular heterogeneity as an underexplored dimension of tumor biology.

### High-MAC TNBC murine tumors exhibit more pericyte covered vasculature and functionally perfused vasculature than Low-MAC models

**Fig. 1:**
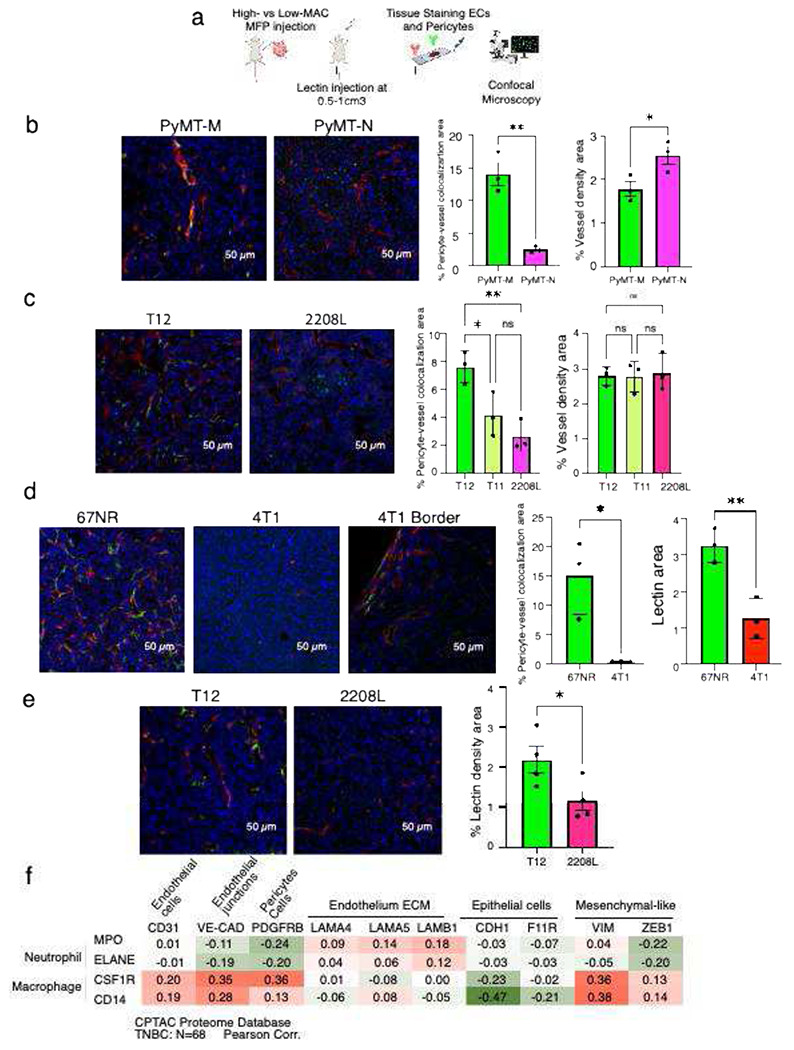
High-MAC TNBC murine tumors exhibit more pericyte covered vasculature and functionally perfused vasculature than Low-MAC models (a) Schematic of experimental workflow. High- and low-MAC triple-negative breast cancer (TNBC) cell lines were orthotopically injected into the mammary fat pad (MFP) of mice. Tumors were collected at 0.5–1 cm^3^, processed for immunofluorescence, and stained for endothelial cells, pericytes, and nuclei (DAPI-Blue). In select experiments, vascular perfusion was assessed by intravenous lectin injection prior to tissue harvest. (b) Representative confocal images of PyMT-M (high-MAC) and PyMT-N (low-MAC) tumors show increased pericyte (yellow) attachment to endothelial cells (red) in PyMT-M. Quantification reveals a significant increase in % pericyte-vessel colocalization area in PyMT-M. Quantification of vessel density. PyMT-N tumors exhibit significantly greater vessel density than PyMT-M (n = 3 mice/group; unpaired two-tailed t-test, p < 0.01**, p < 0.05, *, ns = not significant) (c) Pericyte-vessel colocalization in p53ko lines: T12 (high-MAC) shows significantly higher pericyte (green) coverage of endothelial cells (red) than 2208L (low-MAC), while T11 (High-MAC) shows an intermediate phenotype. Quantification of vessel density, no significant differences are observed among p53ko lines (n = 3 mice/group; unpaired two-tailed t-test, p < 0.01**p < 0.05 * , ns = not significant). (d) Representative images of 67NR (high-MAC) and 4T1 (low-MAC) tumors show increased lectin (red) and pericyte (green) colocalization in 67NR. Border zones of 4T1 tumors show partial perfusion. Quantification indicates significantly higher pericyte-vessel coverage in 67NR. (n = 3 mice/group; unpaired two-tailed t-test, p < 0.05*) (e) Lectin perfusion in p53ko lines: T12 tumors exhibit significantly greater lectin area than 2208L. lectin (red) and pericyte (green) (n = 4 mice/group; unpaired two-tailed t-testp < 0.05*). (f) Heatmap showing Pearson correlation coefficients between myeloid cell markers and vascular/mesenchymal/epithelial markers in TNBC samples from the CPTAC proteomic dataset (n = 68). Macrophage markers (CSF1R, CD14) positively correlate with endothelial and mesenchymal markers and negatively with epithelial markers (e.g., CDH1), suggesting macrophage-driven vascular normalization and mesenchymal transition.

To examine how tumor vasculature varies across distinct immune microenvironments in TNBC, we used syngeneic mouse models previously characterized as High-MAC (PyMT-M, T12/11, 67NR) or Low-MAC (PyMT-N, 2208L, 4T1), the latter also displaying higher neutrophil infiltration [10–12]. We paired these tumors based on their oncogenesis mechanisms and genetic background (Table 1). Vasculature staining (CD31 and NG2) was performed ex vivo on fixed tumor sections, while functional perfusion was assessed in vivo using fluorescent lectin labeling prior to sacrifice [22]. Tumors were subsequently analyzed by confocal microscopy to quantify vessel morphology and pericyte coverage across models (Figure 1a).

Confocal imaging revealed striking differences in vessel architecture between High-MAC (PyMT-M, T12) and Low-MAC (PyMT-N, 2208L) tumors (Figure 1b–c). High-MAC tumors displayed more continuous, pericyte-associated vessels, whereas Low-MAC tumors contained fragmented, irregular networks with sparse pericyte coverage. Quantitative analysis confirmed significantly higher pericyte–vessel colocalization in PyMT-M compared with PyMT-N tumors, and in T12 compared with 2208L, with T11 showing partial normalization. These findings indicate that macrophage-enriched TNBC tumors exhibit increased pericyte–vessel association, consistent with a trend toward more normalized vasculature. Interestingly, PyMT-N tumors had significantly higher vessel density compared to PyMT-M, no significant differences in vessel density were observed among the p53-null lines.

To directly assess vascular functionality, we performed lectin perfusion assays in vivo (Figure 1d). Lectin signal was markedly stronger in high-MAC tumors. In 67NR tumors, lectin penetrance and pericyte coverage were higher than in 4T1 tumors, which showed perfusion only at tumor margins. Consistent with this, T12 tumors exhibited significantly more lectin-positive vasculature than 2208L tumors (Figure 1e), further supporting a more functional vasculature in the high macrophage microenvironment.

To determine whether the vascular patterns observed in our murine TNBC models are reflected in human tumors, we analyzed proteomic data from TNBC patients in the CPTAC [23] cohort (Figure 1f). We observed that macrophage markers (CSF1R, CD14) positively correlated with endothelial (CD31), endothelial junction (VE-CAD), and pericyte-associated (PDGFRB) proteins, whereas they showed negative associations with the epithelial marker CDH1 and positive trends with mesenchymal markers such as VIM. In contrast, neutrophil markers (MPO, ELANE) did not show comparable correlations with vascular components. These findings suggest that macrophage abundance in human TNBC is linked to enhanced expression of vascular and mesenchymal-associated proteins, supporting the association between macrophage-enriched TME and vascular normalization status observed in our mouse models.

### Macrophage abundance is associated with distinct endothelial cell states and vascular programs in murine TNBC

**Fig. 2:**
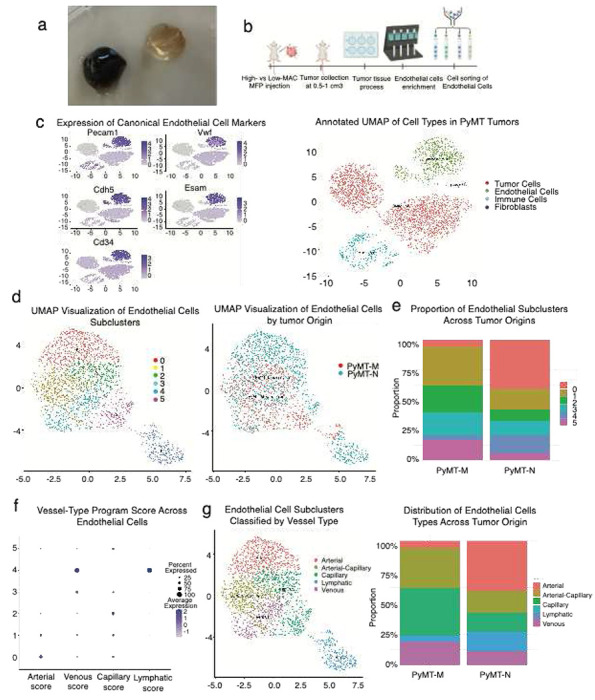
Macrophage abundance is associated with distinct endothelial cell states and vascular programs in murine TNBC (a) Representative image of PyMT-M (left) and PyMT-N (right) tumors. The PyMT-M specimen appears darker and blood-rich, whereas the PyMT-N specimen exhibits a paler, less blood-rich appearance. (b) Experimental workflow for isolating endothelial cells from High-MAC (PyMT-M) and Low-MAC (PyMT-N) murine TNBC tumors. Tumor cells were orthotopically injected into the mammary fat pad (MFP), and tumors were collected at comparable sizes. Following enzymatic dissociation, endothelial cells were enriched and fluorescence-activated cell sorted (FACS) prior to single-cell RNA sequencing analysis. (c) Expression of canonical endothelial cell markers (Pecam1, Vwf, Cdh5, Esam, Cd34) across the UMAP confirms endothelial identity and purity of the population selected for downstream analyses (left). UMAP visualization of single-cell RNA-sequencing data from combined PyMT-M and PyMT-N tumors, showing major cellular compartments including tumor, endothelial, and immune populations (right). (d) UMAP visualization of endothelial cells combined from PyMT-M and PyMT-N tumors, showing six transcriptionally distinct subclusters identified by unsupervised clustering (left). These subclusters represent diverse endothelial phenotypes that are distributed across both tumor types, revealing overlapping yet model-specific vascular populations (right). (e) Proportion of endothelial subclusters identified in previous UMAP. Bar plot showing the relative abundance of each endothelial cell subcluster across tumor origins. (f) Dot plot showing enrichment scores for vessel-type–specific transcriptional programs (arterial, venous, capillary, and lymphatic) across the endothelial subclusters identified in PyMT tumors. Each dot represents the average expression and percentage of cells expressing genes characteristic of each vascular subtype. (g) UMAP visualization of endothelial subclusters classified according to vessel-type gene expression programs (arterial, arterial-capillary, capillary, venous, and lymphatic), showing distinct spatial segregation of vascular identities within the endothelial population. Bar plot displaying the relative distribution of each endothelial subtype across PyMT-M and PyMT-N tumors.

To further investigate the vascular differences observed across murine TNBC models, we focused on the PyMT series, in which High-MAC (PyMT-M) and Low-MAC (PyMT-N) tumors exhibited the most pronounced visual contrast. PyMT-M tumors consistently appeared darker and bloodier than PyMT-N tumors, which were paler and less vascularized in appearance [24] (Figure 2a). These macroscopic differences suggested underlying variation in vascular architecture and composition between the two immune contexts. To define these differences at the cellular level, we performed single-cell RNA sequencing of ECs isolated [25] from both PyMT tumors (Figure 2b). To confirm the identity of endothelial cells within the single-cell dataset, we visualized the expression of canonical endothelial markers, including Pecam1, Vwf, Cdh5, Esam, and Cd34 [26] (Figure 2c). UMAP projection showed that ECs from both groups largely overlapped but exhibited distinct local enrichments, indicating transcriptional divergence associated with macrophage abundance. Unbiased clustering identified six endothelial subclusters representing distinct vascular states (Figure 2d). Comparison of endothelial subcluster composition between PyMT-M and PyMT-N tumors revealed distinct shifts in vascular cell proportions. PyMT-M vasculature was enriched for clusters C1, C2, C3, and C5, whereas clusters C0 and C4 predominated in PyMT-N tumors (Figure 2e).

To better understand the biology underlying different clusters, we re-performed the clustering analysis using prior knowledge of canonical marker genes of different endothelial cell subtypes. This analysis revealed transcriptional programs corresponding to arterial, capillary, venous, and lymphatic [27–29] (Figure f-g). Comparison of EC subtype proportions across PyMT-M and PyMT-N tumors demonstrated marked compositional differences (Figure 2h). PyMT-M (High-MAC) tumors were enriched for venous and capillary ECs, whereas PyMT-N (Low-MAC) tumors contained higher fractions of arterial and lymphatic ECs. The relative depletion of lymphatic endothelium in PyMT-M tumors may exacerbate intravascular accumulation of blood, as impaired lymphatic drainage limits fluid clearance from leaky vessels [30]. PyMT-M tumors favor vascular states associated with stabilization (venous), while PyMT-N tumors are skewed toward immature, sprouting arterial-like programs (arteries). These findings suggest that High- and Low-MAC TNBC microenvironments are associated with distinct endothelial ecosystems, potentially reflecting differences in vessel stabilization, maturation, and type of immune trafficking.

### Endothelial cells in High-MAC TNBC tumors exhibit inflammatory and interferon-associated transcriptional programs

**Fig. 3:**
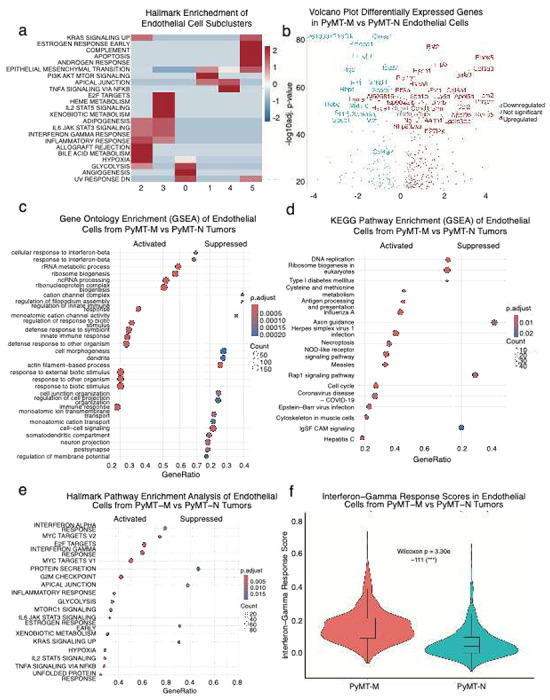
Endothelial cells in High-MAC TNBC tumors exhibit inflammatory and interferon-associated transcriptional programs (a) Heatmap displaying Hallmark pathway enrichment scores across endothelial cell subclusters identified in murine PyMT tumors. Each pathway’s enrichment was calculated based on the top marker genes for each subcluster using the MSigDB Hallmark gene sets. Red and blue colors indicate relative pathway activation or suppression, respectively, after z-score normalization across clusters. Pathway enrichment was assessed using a hypergeometric test (ClusterProfiler), with Benjamini–Hochberg correction for multiple comparisons; only pathways with FDR < 0.05 are shown. (b) Volcano plot showing differentially expressed genes between endothelial cells isolated from PyMT-M vs PyMT-N tumors. Each point represents a single gene, with the x-axis corresponding to log2 fold-change (log2FC) and the y-axis to −log10 adjusted p-value. Genes significantly upregulated in PyMT-M endothelium are shown in red, and those downregulated are shown in blue, while nonsignificant genes are shown in gray. Differential expression was assessed using the MAST test implemented in Seurat, controlling for nFeature_RNA and mitochondrial content as covariates. P-values were adjusted for multiple testing using the Benjamini–Hochberg method; genes with adjusted p-value < 0.05 and |log2FC| ≥ 0.5 were considered significant (c) Hallmark pathway enrichment analysis of endothelial cells isolated from PyMT-M versus PyMT-N tumors. Each point represents a significantly enriched Hallmark pathway, with color indicating the adjusted p-value and point size corresponding to the number of genes contributing to enrichment. Enrichment scores were calculated using Gene Set Enrichment Analysis (GSEA) based on ranked differential expression results between the two endothelial populations. Statistical significance was determined using a permutation-based enrichment test as implemented in the ClusterProfiler R package, with Benjamini–Hochberg correction for multiple testing. Pathways with adjusted p-value < 0.05 were considered significantly enriched. (d) Gene Ontology (GO) enrichment analysis of endothelial cells from PyMT-M versus PyMT-N tumors. The figure displays significantly enriched biological processes identified by Gene Set Enrichment Analysis (GSEA). Each dot represents an individual GO term, with dot size indicating the number of genes associated with that term and color corresponding to the adjusted p-value. Enrichment scores were computed based on ranked differential expression values, and significance was assessed using the permutation-based GSEA test as implemented in the ClusterProfiler R package. Multiple testing correction was applied using the Benjamini–Hochberg method and GO terms with adjusted p-value < 0.05 were considered significantly enriched. (e) KEGG pathway enrichment analysis of endothelial cells from PyMT-M versus PyMT-N tumors. The figure presents pathways significantly enriched in each endothelial population, as determined by Gene Set Enrichment Analysis (GSEA). Each dot represents an individual KEGG pathway, with dot size corresponding to the number of genes contributing to enrichment and color indicating the adjusted p-value. Statistical enrichment was assessed using the ClusterProfiler R package with a permutation-based approach, and multiple testing correction was performed using the Benjamini–Hochberg method. Pathways with adjusted p-value < 0.05 were considered significant. (f) Interferon-γ response scores in endothelial cells from PyMT-M versus PyMT-N tumors. Violin plots show the distribution of interferon-γ response scores across endothelial cells derived from each tumor type. Scores were computed based on the average expression of interferon-stimulated gene sets curated from MSigDB Hallmark pathways. Statistical significance between groups was assessed using a two-sided Wilcoxon rank-sum test, yielding p = 3.30 × 10^−111^.

To assess how these transcriptional states were distributed among endothelial subclusters, we analyzed hallmark pathway enrichment across the six EC populations identified previously. PyMT-M vasculature was enriched for clusters C1, C2, C3, and C5, whereas clusters C0 and C4 predominated in PyMT-N tumors. Clusters 1 displayed PI3K-AKT-MTOR SIGNALING, apical junction and epithelial to mesenchymal transition suggesting actively remodeling. Cluster 2 and 3 displayed interferon-high and inflammatory signaling, indicating heightened immune communication within the vascular niche. Cluster 5 showed enrichment for apoptosis, potentially reflecting hemorrhagic features, in line with the bloody phenotype observed in PyMT-M tumors. Cluster 0 exhibited an angiogenic–glycolytic signature and Cluster 4 was defined by apical junction and TNFA signaling via NFKB, representing also a pro-inflammatory yet barrier-modulating endothelial population (Figure 3a). Together, these findings indicate that macrophage-enriched TNBC tumors harbor endothelial populations transcriptionally primed for immune communication and cytokine responsiveness, whereas non-macrophage-enriched tumors maintain endothelial programs oriented toward structural maintenance and angiogenic remodeling.

Differential expression analysis revealed that PyMT-M ECs upregulated genes associated with inflammation linked to monocyte and macrophage recruitment including *Bst2*, Ifi27l2a, Lgals3, Ccl2 and Hsp90aa1 [31–35], whereas PyMT-N ECs expressed higher levels of structural and vessel identity genes such as Vwf, Sparcl1, Fn1, and Col4a2 [36–37]. Additional PyMT-M–enriched genes, including Col6a1, Aebp1, Emp3 [38–39], reflected matrix remodeling, metabolic activation, and angiogenic signaling, while PyMT-N ECs expressed genes tied to endothelial remodeling and contractile apparatus such as Robo1 and Sema6a [40–41] (Figure 3b).

Gene set enrichment analysis (GSEA) using MSigDB Hallmark pathways identified broad activation of immune and stress response programs in PyMT-M ECs, including interferon-α and interferon-γ responses, inflammatory signaling (IL6–JAK–STAT3, IL2–STAT5), and TNF–NFκB signaling (Figure 3c). These cells also displayed enrichment of E2F and MYC target gene sets, G2M checkpoint, and mTORC1 signaling, reflecting proliferative and metabolically active endothelium. In contrast, structural and junctional pathways such as Apical Junction were relatively suppressed and KRAS signaling.

Complementary GO-based enrichment further supported this pattern, PyMT-M tumors promote an immune-stimulated, biosynthetically active endothelial phenotype, whereas non-macrophage-enriched PyMT-N tumors sustain a structurally organized and quiescent vasculature phenotype. Upregulated pathways included response to interferon-β, innate immune response, defense response to symbiont, and response to external biotic stimulus, reflecting strong activation of antiviral and cytokine-mediated signaling, identifying upregulation of innate and antiviral immune responses, including response to interferon-β, defense response to virus, and regulation of viral replication (Figure 3d).

Additionally, GSEA of KEGG pathways revealed that endothelial cells from PyMT-M tumors were enriched for pathways associated with viral infection, immune activation, and cell-cycle regulation, including hepatitis C, Epstein–Barr virus infection, herpes simplex virus 1 infection, measles, influenza A, coronavirus disease (COVID-19), and antigen processing and presentation. Many of these pathways share components of innate immune and interferon signaling, highlighting broad antiviral and inflammatory activation in macrophage-enriched vasculature. Additional upregulated pathways, such as NOD-like receptor signaling, DNA replication, ribosome biogenesis, and cysteine and methionine metabolism, suggest increased proliferation, biosynthetic activity, and metabolic reprogramming in response to stress and inflammation (Figure 3e). Together, these findings indicate that macrophage-enriched tumors promote transcriptional reprogramming of endothelial cells toward a pro-inflammatory and proliferative state, whereas non-macrophage-enriched tumors maintain a more homeostatic endothelial signature.

Consistent with these signatures and in line with our previous publication [20] demonstrating a connection between interferon-γ signaling and vascular normalization, ECs from PyMT-M tumors displayed significantly higher interferon-γ response scores compared with PyMT-N ECs (Figure 3d–e), confirming transcriptional activation of immune-responsive programs at the single-cell level.

### Human TNBC recapitulates macrophage-associated vascular heterogeneity observed in murine models

**Fig. 4:**
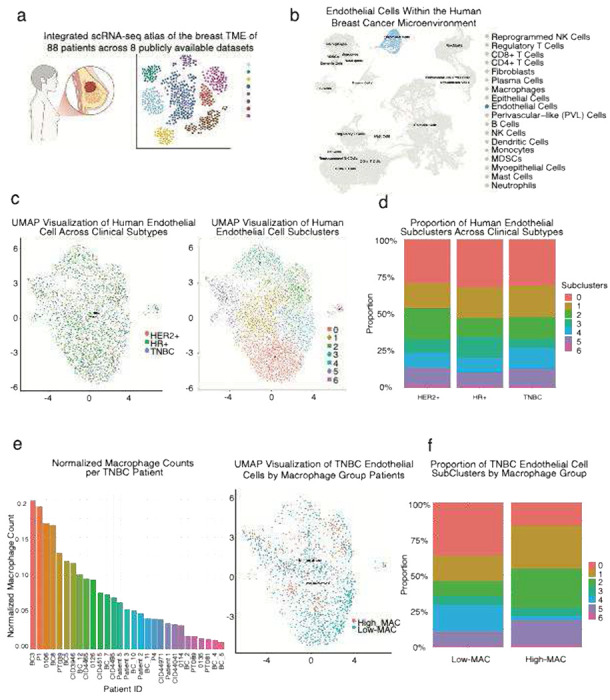
Human TNBC recapitulates macrophage-associated vascular heterogeneity observed in murine models (a) Integrated single-cell RNA sequencing (scRNA-seq) atlas of the breast tumor microenvironment (TME) across 88 patients from eight publicly available datasets. (b) UMAP projection of the integrated scRNA-seq atlas highlighting endothelial cells (ECs) as a distinct transcriptional cluster among diverse tumor-associated cell populations. Each point represents a single cell, color-coded by cell type annotation. (c) UMAP visualization of human endothelial cells across clinical breast cancer subtypes. (Left) Endothelial cells (ECs) from all tumors colored by clinical subtype—triple-negative breast cancer (TNBC), HER2+, and hormone receptor–positive (HR) . (Right) Unsupervised clustering of ECs identifies seven transcriptionally distinct endothelial subclusters. (d) Proportion of human endothelial subclusters across clinical breast cancer subtypes. Bar plot showing the relative abundance of the seven endothelial subclusters across hormone receptor–positive (HR^+^), HER2-positive (HER2^+^), and triple-negative breast cancer (TNBC) tumors. (e) Macrophage abundance and associated endothelial distribution across TNBC patients. (Left) Normalized macrophage counts per TNBC total cell count per patient. (Right) UMAP visualization of endothelial cells colored by macrophage group demonstrates transcriptional differences in vascular populations between High-MAC and Low-MAC tumors. (f) Proportion of TNBC endothelial subclusters across macrophage abundance groups. Stacked bar plot showing the relative distribution of the seven endothelial subclusters (0–6) between High-MAC and Low-MAC TNBC tumors.

To extend our findings from murine TNBC models to human disease, we analyzed an integrated scRNA-seq atlas of the breast TME constructed from eight publicly available datasets [21] (Figure 4a). Endothelial cells formed a distinct transcriptional cluster, underscoring the presence of a well-defined vascular compartment within the human breast cancer TME. This integrated atlas provided the foundation to investigate how macrophage abundance and immune context potentially shape endothelial heterogeneity in human TNBC. (Figure 4b).

We first examined how vascular populations vary across all clinical subtypes. Endothelial cells were present in tumors from all major subtypes: hormone receptor–positive (HR+), HER2+, and triple-negative breast cancer (TNBC) (Figure 4c, left). Unsupervised clustering revealed seven transcriptionally distinct endothelial subclusters (Figure 4c, right). When stratified by clinical subtype, endothelial subcluster composition varied modestly across HER2+, HR+, and TNBC tumors (Figure 4d). These results indicate that endothelial cell heterogeneity is a conserved feature of human breast cancer.

To assess immune heterogeneity within the human TNBC cohort, we quantified macrophage representation per patient based on normalized single cell counts (Figure 4e). Macrophage abundance varied substantially across tumors, with some cases displaying a high proportion of macrophages among all tumor-infiltrating cells, while others exhibited sparse macrophage infiltration. This variation provided a rationale to stratify TNBC patients into High-MAC and Low-MAC groups, mirroring the immune states defined in our murine models. These groups were subsequently used to explore how immune composition associates with endothelial heterogeneity and vascular phenotypes in human TNBC.

We next examined whether endothelial composition varies with macrophage infiltration in human TNBC. Patients stratified into High-MAC and Low-MAC groups showed clear differences in endothelial subcluster distribution (Figure 4f). High-MAC tumors were enriched in subclusters 1, 2, and 5, whereas Low-MAC tumors contained higher proportions of subclusters 0 and 4; and subclusters 3 and 6 were comparably represented across both groups. The most striking observation emerged when comparing the variation in endothelial composition across macrophage groups. The variation in endothelial composition between macrophage groups was substantially greater than that observed across clinical subtypes, underscoring immune context, rather than tumor intrinsic subtype, as a dominant driver of endothelial heterogeneity in TNBC. These results support a conserved association between macrophage abundance and vascular heterogeneity across both mouse and human tumors.

### High-MAC human TNBC tumors exhibit endothelial activation marked by immune signaling and extracellular matrix remodeling but correlates with less T cell infiltration

**Fig. 5:**
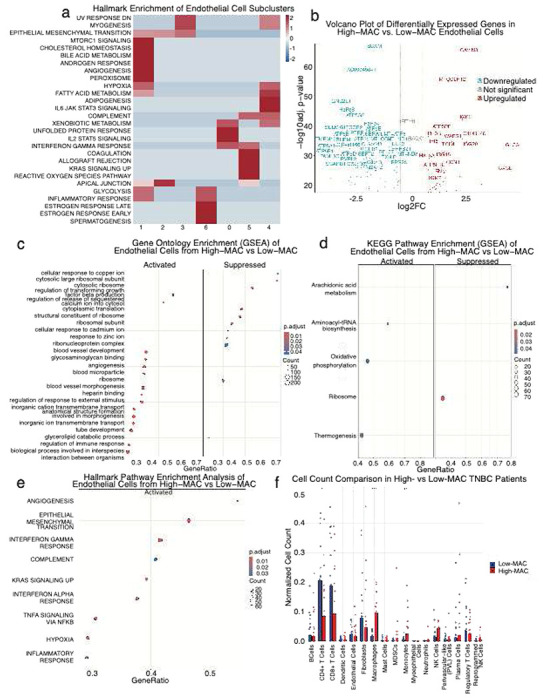
High-MAC human TNBC tumors exhibit endothelial activation marked by immune signaling and extracellular matrix remodeling but correlates with less T cell infiltration (a) Heatmap displaying Hallmark pathway enrichment scores across endothelial cell subclusters identified in human TNBC. Each pathway’s enrichment was calculated based on the top marker genes for each endothelial subcluster using the MSigDB Hallmark gene sets (human version). Red and blue colors indicate relative pathway activation or suppression, respectively, after z-score normalization across clusters. Pathway enrichment was assessed using a hypergeometric test (ClusterProfiler), with Benjamini–Hochberg correction for multiple comparisons; only pathways with FDR < 0.05 are shown. (b) Volcano plot showing differentially expressed genes between endothelial cells isolated from High-MAC vs Low-MAC human TNBC tumors. Each point represents a single gene, with the x-axis corresponding to log2 fold-change (log2FC) and the y-axis to −log10 adjusted p-value. Genes significantly upregulated in High-MAC endothelium are shown in red, and those downregulated are shown in blue, while nonsignificant genes are shown in gray. Differential expression was assessed using the MAST test implemented in Seurat, controlling for nFeature_RNA and mitochondrial content as covariates. P-values were adjusted for multiple testing using the Benjamini–Hochberg method; genes with adjusted p-value < 0.05 and |log2FC| ≥ 0.5 were considered significant (c) Gene Set Enrichment Analysis (GSEA) of Hallmark pathways comparing endothelial cells from High-MAC versus Low-MAC human TNBC tumors. Each point represents a significantly enriched Hallmark pathway, with color indicating the adjusted p-value and point size corresponding to the number of genes contributing to enrichment. Enrichment scores were calculated using Gene Set Enrichment Analysis (GSEA) based on ranked differential expression results between the two endothelial populations. Statistical significance was determined using a permutation-based enrichment test as implemented in the ClusterProfiler R package, with Benjamini–Hochberg correction for multiple testing. Pathways with adjusted p-value < 0.05 were considered significantly enriched. (d) GSEA of Gene Ontology (GO) terms highlighting biological processes enriched in endothelial cells from High-MAC versus Low-MAC tumors. ach dot represents an individual GO term, with dot size indicating the number of genes associated with that term and color corresponding to the adjusted p-value. Enrichment scores were computed based on ranked differential expression values between endothelial cells from High-MAC and Low-MAC tumors, and significance was assessed using the permutation-based GSEA test as implemented in the ClusterProfiler R package. Multiple testing correction was applied using the Benjamini–Hochberg method and GO terms with adjusted p-value < 0.05 were considered significantly enriched. (e) KEGG pathway enrichment analysis of endothelial cells from high- versus low-MAC human TNBC tumors. The figure presents pathways significantly enriched in each endothelial population, as determined by Gene Set Enrichment Analysis (GSEA). Each dot represents an individual KEGG pathway, with dot size corresponding to the number of genes contributing to enrichment and color indicating the adjusted p-value. Statistical enrichment was assessed using the ClusterProfiler R package with a permutation-based approach, and multiple testing correction was performed using the Benjamini–Hochberg method. Pathways with adjusted p-value < 0.05 were considered significant. (f) Bar plots show the normalized abundance of major stromal and immune cell populations in tumors from High-MAC and Low-MAC TNBC patient groups. Each point represents an individual patient, and normalization was performed relative to the total number of cells per patient. Statistical significance between groups for each cell type was assessed using two-sided Wilcoxon rank-sum tests, with asterisks indicating significant differences (*p < 0.05). n = 29, High-MAC = 14 and Low-MAC n = 15.

To better define the phenotype of each endothelial subcluster in human TNBC tumors, we performed Hallmark pathway enrichment analysis across the seven endothelial populations identified previously. Clusters 0 and 4, predominant in Low-MAC tumors, exhibited stress-adaptive, barrier-stabilizing, yet immune-responsive endothelial programs, suggesting maintenance of vascular integrity under mild inflammatory stress. In contrast, clusters 1, 2, and 5, enriched in high-MAC tumors, displayed transcriptional hallmarks of angiogenic activation, metabolic engagement, inflammation, and vascular remodeling, consistent with a more reactive and remodeling-prone vasculature (Figure 5a).

Differential expression analysis of endothelial cells from High- versus Low-MAC TNBC tumors revealed distinct transcriptional programs (Figure 5b). Endothelial cells from High-MAC tumors upregulated genes linked to immune activation and interferon signaling (*CXCL11, ISG20, IFI6 and ISG15*) [42–45], consistent with our mouse data. Several extracellular matrix–associated genes, including *FN1* (*Fibro*necti*n*), CCN1, TGM2 [46–48] were also elevated, indicating active vascular remodeling. High-MAC ECs also showed mitochondrial components (ATP5PF, ATP5F1E, MTCO2P12) [49–50] suggesting a metabolically engaged phenotype.

Conversely, ECs from Low-MAC tumors showed increased expression of mitochondrial oxidative-phosphorylation and ATP synthase complex genes (*MT-CO2, MT-ND3, ATP5G2, ATP5A1, ATP5B, ATP5D, ATP5E, ATP5L, ATP5F1, MT-ATP8, MT-ND4L)* [49–51], genes involved in protein synthesis and ribosome structure *(EF2, RPS28, RPL37, H2AFZ)* [52–54]. They also show higher structural adhesion molecules along with angiogenic mediators expression (*BCAM, SCARB1, SEPT2, SEPT7, PTRF (Cavin1), GNB2L1 (RACK1))* [55–60]. These transcriptional patterns suggest that endothelial cells in non-macrophage-enriched tumors adopt a more oxidative phosphorylation driven, protein-synthetic, and junctional stable state, whereas macrophage-enriched tumors promote an inflamed, remodeling endothelium characterized by interferon-stimulated and matrix-interactive phenotypes, likely relying more heavily on glycolytic metabolism.

Hallmark pathway analysis revealed that ECs from High-MAC tumors exhibited broad activation of inflammatory and stress-response programs. Upregulated pathways included TNFα signaling via NFκB, inflammatory response, and interferon-α/γ responses, consistent with an immune-activated and cytokine-responsive endothelium. Concurrent enrichment of hypoxia, KRAS signaling, and epithelial–mesenchymal transition (EMT) pathways indicated metabolic reprogramming and endothelial plasticity, reflecting an adaptive response to the hypoxic and inflammatory tumor microenvironment (Figure 5c).

Gene Ontology (GO) enrichment analysis revealed distinct functional specialization between endothelial cells from High- and Low-MAC tumors. High-MAC ECs upregulated pathways related to immune activation, vascular remodeling, and intercellular communication, including regulation of immune response, angiogenesis, blood vessel morphogenesis, and transforming growth factor β (TGF-β) signaling. These programs reflect an inflammatory and remodeling-prone vascular phenotype, likely shaped by paracrine cues from macrophage-enriched tumor microenvironments. In contrast, Low-MAC ECs showed enrichment of ribosomal biogenesis, ribonucleoprotein complex formation, and metal ion response pathways, indicative of a translationally active. Together, these findings suggest that macrophage abundance associates to a shift toward a pro-inflammatory, angiogenic, and stress-adaptive phenotype in tumor vasculature. (Figure 5d).

Additionally, KEGG pathway analysis revealed distinct metabolic adaptations between both groups. High-MAC ECs upregulated oxidative phosphorylation and thermogenesis, indicating increased mitochondrial activity and energy expenditure, consistent with an activated, immune responsive vasculature. Conversely, Low-MAC ECs showed enrichment of ribosome and aminoacyl-tRNA biosynthesis pathways, reflecting enhanced translational capacity and protein synthesis characteristic of a more engaged in maintenance rather than inflammatory activation (Figure 5e).

To examine how macrophage heterogeneity shapes the broader immune landscape, we compared immune and stromal cell composition between High- and Low-MAC TNBC patient groups (Figure 5f). Low-MAC tumors exhibited significantly higher proportions of CD4^+^ T cells and a trend toward increased CD8^+^ T cells, suggesting greater lymphocytic infiltration in these tumors. In contrast, High-MAC tumors contained elevated frequencies of monocytes, consistent with a myeloid-enriched microenvironment. TAM have shown that can physically and molecularly restrict T-cell infiltration which could explain this phenotype [61]. These results indicate that macrophage-enriched TNBC tumors are associated with myeloid-dominant immune contexts, whereas non-macrophage-enriched tumors show enhanced T-cell presence, potentially reflecting differential immune–vascular dynamics across TNBC subtypes.

## Discussion

This study reveals that macrophage abundance is closely associated with vascular heterogeneity in triple-negative breast cancer (TNBC). Across murine TNBC models, macrophage-enriched tumors exhibited more normalized vasculature, characterized by greater pericyte coverage and functional perfusion, whereas nonmacrophage-enriched tumors displayed denser but immature and poorly perfused vessels. Single-cell RNA sequencing showed that these vascular features coincide with distinct endothelial transcriptional programs: macrophage-enriched tumors were enriched for venous endothelial states expressing immune responsiveness and extracellular-matrix–related signatures, while non-macrophage-enriched tumors displayed arterial, and lymphatic and endothelial homeostatic phenotype.

In human TNBC, macrophage abundance similarly associated with vascular diversity, linking myeloid-enriched microenvironments to specific endothelial phenotypes such as inflamed, and remodeling-prone but inversely correlating with T-cell infiltration, consistent with known macrophage-associated physical barriers to lymphocyte entry [61]. Notably, the variation in endothelial composition associated with macrophage abundance exceeded that observed across intrinsic breast cancer subtypes, underscoring immune context as a dominant correlate of vascular heterogeneity.

While largely correlative, these results highlight that TNBC should be viewed as a collection of distinct immunovascular ecosystems rather than a single entity. Tumor vasculature is not a binary state of “normal” versus “abnormal,” but rather exists along a spectrum of phenotypes that vary with immune context. Defining how these immune and vascular compartments interact will be critical for understanding therapeutic variability and for developing strategies that address the layered heterogeneity of TNBC.

## Methods

### Mice Studies.

All animal experiments were conducted in accordance with a protocol approved by Institutional Animal Care and Use Committee of Baylor College of Medicine. The study is compliant with all relevant ethical regulations regarding animal research. Female animals of 6–8 weeks of age were used as the recipients of cell line transplantation. BALB/cAnNHsd (RRID: IMSR_ENV:HSD-047), C57BL/6NHsd (RRID: IMSR_ENV:HSD-044) mice were purchased from Envigo and either directly used for experiments or bred in our facilities.

### Breast tumour models and transplantation.

Tumour models include T11 (BALB/c, p53-null tumour, RRID: not available), 2208L (BALB/c, p53-null tumour, RRID: not available) T12 (BALB/c,p53-null tumour, RRID: not available), PyMT-M and -N (B6, MMTV-PyMT sub-lines, RRID: not available), 4T1 (BALB/c, spontaneous, RRID: CVCL_0125) and 67NR (BALB/c, spontaneous, RRID: CVCL_9723). Cell lines were derived from above models and maintained as described in the ‘Cell lines and cell culture’ section. For inoculation into animals, cells were collected from culture with 0.25% trypsin (HyClone), washed with PBS (Lonza), counted, resuspended in 1:1 solution of PBS and Matrigel (Pheno Red-free and growth factor reduced; BD Biosciences), and injected into the fourth mammary fat pad. Tumour models include (genetic background and cell numbers used for transplantation are indicated in parentheses): 4T1 (BALB/c, 0.05 × 10^6 cells), 67NR (BALB/c, 1 × 10^6 cells), T11 (BALB/c, 0.2–0.25 × 10^6 cells), T12 (BALB/c, 0.2–0.25 × 10^6 cells), 2208 (BALB/c, 0.12 × 10^6 cells), PyMT-M (B6, 0.12 × 10^6 cells), PyMT-N (B6, 0.12 × 10^6 cells).Mammary fat pad transplantation and injection were performed using the same procedures as our previous studies [62].

### Cell lines and cell culture.

All cell lines were cultured in DMEM/high glucose medium (HyClone). All media contained 10% FBS (Thermo Fisher Scientific), 1% Corning^™^ Antibiotic-Antimycotic Solution (Corning-VWR), except that for 67NR, which was further supplemented with NEAA (Life Technologies). All cells were grown in a humidified incubator at 37 °C, with 5% CO_2_.

### Human Patient Samples.

Publicly available human breast tumor single-cell RNA sequencing data were obtained from the *Cell Reports Medicine* study by Xu et al. (2024). This integrated dataset comprises 236,363 cells from 88 patients and 119 biopsy samples representing all major breast cancer subtypes and provides curated cell-type annotations and metadata. Data were accessed via the Gene Expression Omnibus (GEO accession: GSE261030) as described in the original publication. For this study, only triple-negative breast cancer (TNBC) samples were included in downstream analyses. Endothelial and immune cell subsets were re-analyzed using the Seurat (v5) workflow for normalization, dimensionality reduction, clustering, and pathway enrichment.

### Isolation of endothelial cells from tumors.

Tumors were mechanically dissociated and enzymatically digested in DMEM containing 10% FBS (Thermo Fisher Scientific), 2 mg/mL collagenase II (Thermo Fisher Scientific), 2 mg/mL dispase II (Sigma-Aldrich), and DNase I (1:200, Sigma-Aldrich) at 37 °C for 60 min with intermittent trituration. Cell suspensions were filtered through 70-μm strainers, subjected to red blood cell lysis (1× RBC buffer, 5 min on ice, Cytek Biosciences), and washed in FACS buffer (PBS, 2% FBS, % Corning^™^ Antibiotic-Antimycotic Solution). Debris was removed using a density gradient (Debris Removal Solution, Miltenyi Biotec), and cells were resuspended in MACS buffer (PBS, 2% FBS, 2 mM EDTA). For magnetic separation, Fc receptors were blocked (anti-CD16/32, 5 min, 4 °C, RRID: AB_2621443) followed by incubation with biotinylated anti-CD45 and anti-Ter119 antibodies (15 min, 4 °C, Tonbo Bioscience, no RRId found). Streptavidin-coated magnetic beads were applied, and unlabeled (CD45^−^/Ter119^−^) cells were collected as the stromal fraction. For positive selection, endothelial populations were isolated using biotinylated anti-CD31 (Miltenyi Biotec, RRID:AB_2814657), and anti-VE-cadherin (CD144, BioLegend RRID:AB_10641138) antibodies, followed by streptavidin bead enrichment and magnetic retention. Tumor sections were stained with Alexa Fluor^®^ 647–conjugated anti-CD144 (VE-cadherin, BioLegend, RRID:AB_10569114) and anti-CD31 (PECAM-1) conjugated to APC-Cy7 (BioLegend, RRID:AB_2860593). Enriched fractions were fluorescence-activated cell sorted (FACS) on a cell sorter to obtain pure endothelial (CD31^+^ CD144^+^) populations. Dead cells were excluded using DAPI (Invitrogen^™^) staining, and single-color and unstained controls were included for compensation. Sorted populations were collected in 2% FBS/PBS and immediately processed for downstream RNA analysis.

### Immunofluorescence staining of OCT-embedded tumor sections.

Frozen tumor sections (10–20 μm thick) embedded in OCT were thawed at room temperature, rehydrated in PBS, and fixed in 10% formalin for 10 min. Autofluorescence was quenched using 0.1 M ammonium chloride in PBS. Sections were permeabilized in PBS containing 0.1–0.5% Triton X-100 (depending on nuclear antigen accessibility) and blocked for 1 h at room temperature in 5% donkey and 5% goat serum prepared in PBS with 0.1% Triton and 2% fish gelatin (PBS-GT). Primary antibodies were diluted in PBS-GT containing 1.5% normal serum and incubated overnight at 4 °C in a humidified chamber. After three 10-min PBS washes, fluorophore-conjugated secondary antibodies (1:200) were applied for 2 h at room temperature, followed by nuclear counterstaining with DAPI (1:1000, Invitrogen^™^). Slides were mounted with ProLong^™^ Gold antifade reagent and imaged on a Zeiss LSM confocal microscope or whole-slide fluorescence scanner. The following primary antibodies were used: anti-CD31 (goat, 1:200, R&D Systems, RRID:AB_2161028) and anti-VE-cadherin (goat, 1:200, R&D Systems, RRID:AB_2077789), anti-NG2 (rabbit, 1:200, Millipore Cat, RRID:AB_11203295). Donkey anti-goat Alexa Fluor 555 secondary antibody (Invitrogen, RRID: AB_2535853) and Alexa Fluor^®^ 647 AffiniPure^®^ Donkey Anti-Rabbit IgG (H+L) secondary antibody (Jackson ImmunoResearch Inc., RRID: AB_2492288) were used at 1:200. For vessel quantification, five random fields per tumor were imaged and analyzed under identical acquisition settings. Images were processed and quantified in Fiji (ImageJ, NIH) using consistent thresholding and region-of-interest parameters across all sample.

### FIJI Image Analyses.

Quantification of vascular normalization parameters was performed using FIJI/ImageJ (RRID:SCR_002285) on confocal images acquired under identical acquisition settings. To calculate pericyte–vessel colocalization, the endothelial (red color) channel was used to define the total vascular area using an automated threshold. The pericyte (green color) channel was then merged with the endothelial channel to generate a yellow colocalization mask representing regions where pericyte and endothelial signals overlapped. The colocalized area (yellow) was measured and expressed as a percentage of the total vascular area (yellow area / total red area × 100), representing the fraction of vessels covered by pericytes. For vessel density, the total vascular area (red channel) was divided by the total image area (in μm^2^) to obtain the percentage of vessel coverage per field.

### In vivo lectin perfusion and vascular labeling.

To visualize functional tumor vasculature, mice were anesthetized and injected retro-orbitally with 200 μL of PBS containing 100 μg Lycopersicon esculentum (tomato) lectin, DyLight 649 (Vector Laboratories). After 10–15 min circulation, mice were transcardially perfused with 10 mL of PBS followed by 10 mL of 10% formalin. The right atrium was opened to permit outflow, and perfusion was continued until the liver blanched, indicating complete blood clearance. Tumors and organs were excised immediately, fixed overnight in 4% paraformaldehyde at 4 °C, and washed three times in PBS. Fixed tissues were either stored in PBS at 4 °C or processed for OCT embedding and immunofluorescence staining. This procedure labels actively perfused vessels in vivo, enabling assessment of vascular functionality and perfusion efficiency across tumor types.

### Bioinformatics analyses.

#### Processing the single-cell RNA sequencing data.

A filtering process was applied to ensure data quality. Cells with fewer than 200 detected genes or exhibiting a deviation of more than 2-fold from the median gene count were excluded. Cells with more than 10% mitochondrial genes were systematically filtered out to minimize mitochondrial gene contamination. We proceeded to normalize each dataset using the NormalizeData function’s default parameters. Following normalization, integration anchors were identified with the FindIntegrationAnchors function using the reciprocal PCA (RPCA) method. The function internally selected the top 2,000 variable features for cross-dataset alignment and batch correction. Anchors were then used to merge the datasets into a single batch-corrected object using the IntegrateData. The integrated assay was set as the default and scaled using ScaleData. Principal component analysis (PCA) was performed on the integrated data using RunPCA, and the first 30 principal components were used to construct a shared nearest-neighbor (SNN) graph with FindNeighbors. Unsupervised clustering was performed using the Louvain algorithm implemented in Seurat’s FindClusters function. To visualize cellular relationships in two-dimensional space, Uniform Manifold Approximation and Projection (UMAP) was computed using RunUMAP.

#### Clustering and cell type identification.

The next phase of the analysis involved clustering the high-quality single-cell data to identify endothelial cells. This was accomplished using the Seurat R package. Upon integration, the data were clustered into 4 distinct cell clusters, each representing a unique population within the samples. Canonical endothelial markers were visualized with FeaturePlot (e.g., Pecam1, Vwf, Cdh5, Esam, Cd34), and immune identity was checked via Ptprc (CD45). Cluster markers were called using FindAllMarkers. Clusters were then manually annotated (e.g., Endothelial, Tumor, Immune, Fibroblast) via RenameIdents, and the labels were stored in metadata. Additionally, further subgroup annotations within endothelial cells, were conducted using the same rigorous methodology to ensure accuracy of the analysis. For the human data, Endothelial cells (ECs) were subset from the full tumor microenvironment, we visualized UMAPs colored by Cell_Type_Annotation == “Endothelial Cells”.

#### Compositional analysis.

Endothelial subcluster composition across tumor origins was summarized as stacked bar plots. Counts and within-sample proportions were computed and displayed as percent with ggplot2.

#### Differential expression and visualization of marker genes.

To identify differentially expressed genes (DEGs) among endothelial cells of Pymt-M and Pymt-N models, differential expression analysis was performed using the FindMarkers function in the Seurat R package. Statistical significance was assessed using the Wilcoxon rank-sum test with Bonferroni correction for multiple comparisons. Genes with an adjusted P-value < 0.05 and |log_2_ fold change| > 0.25 were considered significantly differentially expressed. The resulting DEGs were visualized using a volcano plot, generated in R with the ggplot2 package. Log_2_ fold change values were plotted on the x-axis, and −log_10_(adjusted P-values) on the y-axis, with significantly upregulated and downregulated genes highlighted in distinct colors to emphasize key transcriptional signatures across endothelial populations.

#### Functional annotation of endothelial subclusters via Hallmark enrichment.

For each subcluster’s top-10 marker set, we ran ORA with clusterProfiler enricher using the Hallmark TERM2GENE mapping (multiple testing by Benjamini–Hochberg). For each enriched term we recorded the adjusted P value (p.adjust) and GeneRatio (hits / set size). To provide a uniform visualization score we computed Score = −log10(FDR). We selected the top K = 10 Hallmark terms per subcluster by Score. We reshaped the Score matrix (pathways × subclusters), removed all-zero rows, row-z-scored values, and ordered rows and columns by Spearman correlation with Optimal Leaf Ordering.

Functional annotation of endothelial cell marker genes was performed through Gene Ontology (GO), Kyoto Encyclopedia of Genes and Genomes (KEGG), and Reactome pathway enrichment analyses. Enrichment analyses were conducted using the clusterProfiler R package (v4.10.0), which enables both overrepresentation analysis (ORA) and gene set enrichment analysis (GSEA) with multiple correction options and organism-specific annotation databases. Mouse gene symbols were converted to Entrez Gene IDs using org.Mm.eg.db to ensure compatibility across databases. GO enrichment was performed across all ontologies (Biological Process, Molecular Function, and Cellular Component) using gseGO and enricher functions in clusterProfiler. Terms with an adjusted P value (Benjamini–Hochberg correction) below 0.05 were considered significantly enriched. For pathway-level annotation, KEGG enrichment was carried out using gseKEGG (organism code “mmu”), while Reactome pathway enrichment was assessed using enricher with the Reactome gene sets provided within the same package. For each analysis, a minimum gene set size of 10 and a maximum of 800 were used to maintain statistical robustness. In parallel, Hallmark gene set enrichment analysis was performed using msigdbr (category “H”), which retrieves curated, nonredundant pathways derived from the Molecular Signatures Database (MSigDB). Pre-ranked GSEA was run using log_2_ fold changes from differential expression results, with p values adjusted by the Benjamini–Hochberg method. The top enriched GO terms and pathways were visualized as dot plots and heatmaps using enrichplot and pheatmap.

#### Program scoring.

An interferon-γ response score was computed per endothelial cell using the Hallmark IFN-γ gene set (mouse) retrieved via msigdbr; genes present in the object were intersected and scored with AddModuleScore (name = “HALLMARK_INTERFERON_GAMMA_RESPONSE”). Group differences (PyMT-M vs PyMT-N) were visualized by FeaturePlot and VlnPlot and formally compared with a two-sided Wilcoxon rank-sum test.

#### Endothelial phenotyping.

To assess vessel-type programs, curated endothelial signatures (ARTERIAL, VENOUS, CAPILLARY, LYMPHATIC) were defined from literature genes, case-matched to symbols in the dataset, and scored in a single AddModuleScore. The resulting columns were renamed to visualized with FeaturePlot and DotPlot across endothelial subclusters. For interpretability, subclusters were also manually mapped to vessel-type labels and used in composition plots.

#### Macrophage normalization and grouping (patient-level).

Within human TNBC, we computed per-patient total cell counts and macrophage counts (Cell_Type_Annotation == “Macrophages”). We added a normalized macrophage fraction (Macrophage_Count / Total_Cell_Count) to metadata and stratified patients into High vs Low macrophage groups by the median normalized fraction. We then propagated these group labels back to endothelial cells using Patient_ID, defining Macrophage_Groups by high macrophage and low macrophage.

### Proteomic analyses.

Proteomic data were obtained from the CPTAC triple-negative breast cancer (TNBC) dataset (n = 68). Log_2_-normalized protein abundance values were used to assess correlations between immune cell markers (CSF1R, CD14, MPO, ELANE) and representative stromal or epithelial proteins, including endothelial (CD31), endothelial junction (VE-CAD), pericyte (PDGFRB), endothelial ECM (LAMA4, LAMA5, LAMB1), epithelial (CDH1, F11R), and mesenchymal-like (VIM, ZEB1) markers. Pearson correlation coefficients were calculated and visualized as heatmaps using R v4.3.1 and ggplot2.

### Statistics and reproducibility.

Data were analyzed with Microsoft Excel functions, Prism 7 software (GraphPad) or the R programming language. Statistical analysis was performed as appropriate for each dataset. Comparisons between two groups were conducted using unpaired two-tailed t-tests for parametric data or two-sided Wilcoxon rank-sum tests for nonparametric single-cell comparisons, as indicated in the figure legends. Correlation analyses in proteomic datasets were performed using Pearson correlation coefficients.For single-cell RNA sequencing data, differential gene expression between groups was assessed using the MAST test implemented in the Seurat package, controlling for gene detection rate (nFeature_RNA) and mitochondrial content as covariates. Multiple testing corrections were applied using the Benjamini–Hochberg false discovery rate (FDR) method, with genes or pathways considered significant at adjusted p < 0.05. Pathway enrichment analyses were performed using ClusterProfiler, employing hypergeometric tests for overrepresentation analysis (ORA) and permutation-based Gene Set Enrichment Analysis (GSEA) for ranked gene lists, using Hallmark, Gene Ontology (GO), and KEGG gene sets from the Molecular Signatures Database (MSigDB). All enrichment results were corrected for multiple comparisons using the Benjamini–Hochberg method (FDR < 0.05). Statistical details (for example, sample size and specific test performed) for each experiment are denoted in the corresponding figure or figure legends. Individual mice (independent batch experiments, different tumour models and different animals) were considered biological replicates. All biologically independent samples were included and combined for statistical analyses. Experimental findings were reliably reproduced. In each experiment, group sizes were determined based on the results of preliminary experiments, and no statistical method was used to predetermine sample size. Data are shown as means ± standard deviation (s.d.) unless otherwise specified. P values lower than 0.05 were considered statistically significant.

### Manuscript preparations.

Portions of this manuscript’s text were refined using Grammarly and a large language model (ChatGPT, OpenAI) to improve grammar, clarity, and readability. All content was reviewed, verified, and edited by the authors to ensure scientific accuracy and originality.

## Figures and Tables

**Table 1.** Classification of syngeneic TNBC mouse models by macrophage abundance, strain background, and oncogenic driver

Tumor model	MAC phenotype	Mouse background	Oncogenic driver/tumor genetics
T11	High-MAC	BALB/c	*p53*-KO
T12	High-MAC	BALB/c	*p53*-KO
67NR	High-MAC	BALB/c	Spontaneous
PyMT-M	High-MAC	C57BL/6	MMTV-PyMT
2208L	Low-MAC	BALB/c	*p53*-KO
4T1	Low-MAC	BALB/c	Spontaneous
PyMT-N	Low-MAC	C57BL/6	MMTV-PyMT

a High-MAC tumors are characterized by macrophage-enriched immune microenvironments, whereas Low-MAC tumors are characterized by non–macrophage-enriched immune microenvironments. These classifications align with prior lab publications [10–12].

## Data Availability

The mouse single-cell RNA sequencing data generated in this study have been deposited in GEO under accession number GEO: GSE314557. Human breast tumor single-cell RNA-seq data were obtained from the publicly available resources provided in the original publication by Xu et al. [21]. All code used for data processing, analysis, and figure generation is available on GitHub at https://github.com/xzhanglab/TNBC_Endothelial_Heterogeneity. Quantified imaging data used in Figures 1 (vessel density, pericyte–vessel colocalization, and lectin perfusion measurements) are provided as supplementary files. Raw microscopy image files are large and therefore not publicly archived but are available from the corresponding author upon reasonable request.
